# Quantifying the annual incidence and underestimation of seasonal influenza: A modelling approach

**DOI:** 10.1186/s12976-020-00129-4

**Published:** 2020-07-10

**Authors:** Zachary McCarthy, Safia Athar, Mahnaz Alavinejad, Christopher Chow, Iain Moyles, Kyeongah Nah, Jude D. Kong, Nishant Agrawal, Ahmed Jaber, Laura Keane, Sam Liu, Myles Nahirniak, Danielle St Jean, Razvan Romanescu, Jessica Stockdale, Bruce T. Seet, Laurent Coudeville, Edward Thommes, Anne-Frieda Taurel, Jason Lee, Thomas Shin, Julien Arino, Jane Heffernan, Ayman Chit, Jianhong Wu

**Affiliations:** 1 Department of Mathematics and Statistics, York University, Toronto, M3J 1P3 Canada; 2 Laboratory for Industrial and Applied Mathematics, York University, Toronto, M3J 1P3 ON Canada; 3 Centre for Disease Modelling, York University, Toronto, M3J 1P3 ON Canada; 4 Fields-CQAM Mathematics for Public Health Laboratory, York University, Toronto, M3J 1P3 ON Canada; 5 University of Alberta, Edmonton, T6G 2R3 AB Canada; 6 University of Guelph, Guelph, N1G 2W1 ON Canada; 7 McMaster University, Hamilton, L8S 4L8 ON Canada; 8 Lunenfeld-Tanenbaum Research Institute, Sinai Health System, Toronto, M5G 1X5 ON Canada; 9 Sanofi Pasteur, Toronto, M2R 3T4 Canada; 10 Department of Molecular Genetics, Toronto, M5S 1A8 ON Canada; 11 Sanofi Pasteur, Lyon, France; 12 University of Manitoba, Department of Mathematics, Winnipeg, R3T 2N2 MB Canada; 13 Leslie Dan School of Pharmacy, University of Toronto, Toronto, M5S 3M2 ON Canada; 14 Sanofi Pasteur, Swiftwater, 18370 PA USA

**Keywords:** Mathematical modelling, influenza, vaccine, evidence synthesis

## Abstract

**Background:**

Seasonal influenza poses a significant public health and economic burden, associated with the outcome of infection and resulting complications. The true burden of the disease is difficult to capture due to the wide range of presentation, from asymptomatic cases to non-respiratory complications such as cardiovascular events, and its seasonal variability. An understanding of the magnitude of the true annual incidence of influenza is important to support prevention and control policy development and to evaluate the impact of preventative measures such as vaccination.

**Methods:**

We use a dynamic disease transmission model, laboratory-confirmed influenza surveillance data, and randomized-controlled trial (RCT) data to quantify the underestimation factor, expansion factor, and symptomatic influenza illnesses in the US and Canada during the 2011-2012 and 2012-2013 influenza seasons.

**Results:**

Based on 2 case definitions, we estimate between 0.42−3.2*%* and 0.33−1.2*%* of symptomatic influenza illnesses were laboratory-confirmed in Canada during the 2011-2012 and 2012-2013 seasons, respectively. In the US, we estimate between 0.08−0.61*%* and 0.07−0.33*%* of symptomatic influenza illnesses were laboratory-confirmed in the 2011-2012 and 2012-2013 seasons, respectively. We estimated the symptomatic influenza illnesses in Canada to be 0.32−2.4 million in 2011-2012 and 1.8−8.2 million in 2012-2013. In the US, we estimate the number of symptomatic influenza illnesses to be 4.4−34 million in 2011-2012 and 23−102 million in 2012-2013.

**Conclusions:**

We illustrate that monitoring a representative group within a population may aid in effectively modelling the transmission of infectious diseases such as influenza. In particular, the utilization of RCTs in models may enhance the accuracy of epidemiological parameter estimation.

## Introduction

The exact number of cases of a disease is complex to capture. Different methods can be used, from epidemiological studies to disease surveillance systems. While data collected routinely for surveillance purposes have the advantage of being readily accessible over a long period of time, they are subject to underestimation. Underestimation is a combination of under-reporting (failure to capture cases that seek care due to underdiagnoses or under-notifications) and under-ascertainment (failure to seek health care) [1]. Symptomatic individuals who seek medical care but are misdiagnosed due to an atypical presentation which does not fit the case definition or to the lack of sensitivity of the laboratory test (under-diagnosis) and/or for which administrative steps may not be taken at the physician’s office to report the case contribute to under-reporting [1]. Also, infected individuals may be asymptomatic or with a mild form of the disease and may not seek healthcare, leading to under-ascertainment. Mathematical modelling may play a role in quantifying the effects of factors contributing to underestimation to assess the true number of influenza illnesses, ultimately to assist in policy development and to evaluate the impact of influenza vaccination.

Passive influenza surveillance systems are not designed to capture all illnesses; however, surveillance data has been utilized to assess under-reporting, underestimation and incompleteness. Statistical modelling has been utilized to quantify under-reporting and underestimation of influenza-associated hospitalizations, morbidity and mortality in the United States (US) and Canada [2–12]. However, assessing the underestimation associated with the true number of influenza illnesses has received less attention. Influenza surveillance data has been utilized to assess underestimation and estimate symptomatic influenza illnesses using mulitiplier methods [13, 14], Bayesian evidence synthesis [15], and dynamic models using ordinary differential equations [16]. In one study, assessments of underestimation were shown to be unreliable when utilizing only virologic surveillance data [16]. Similarly, a noted contributor of error of symptomatic illness estimates was the quality of sentinel influenza-like illness (ILI) data, which is subject to both overestimation and underestimation [15]. A method to assess underestimation of influenza illnesses utilizing a reliable data source, such as active surveillance, may enhance the ability to quantify the true underestimation and the number of influenza illnesses. In the present study, we develop a framework to utilize data from a closely monitored group whose epidemiological status is subject to minimal uncertainty. In particular, we utilize a randomized-controlled trial (RCT) designed to minimize the effects of underestimation and completely capture participant’s illnesses through active surveillance and systematic testing [17].

A recent vaccine RCT, which assessed the efficacy of a high-dose (HD) influenza vaccine compared to a standard-dose (SD) influenza vaccine, presents an opportunity to explore a new method using mathematical modelling to correct for underestimation in national virological influenza surveillance, ultimately to infer the number of symptomatic influenza illnesses. In particular, we establish a parameter estimation technique based on a mechanistic disease transmission model to assess the underestimation and symptomatic illnesses in the US and Canada during the 2011-2012 and 2012-2013 influenza seasons.

## Methods

### Data sources

**Fig. 1:**
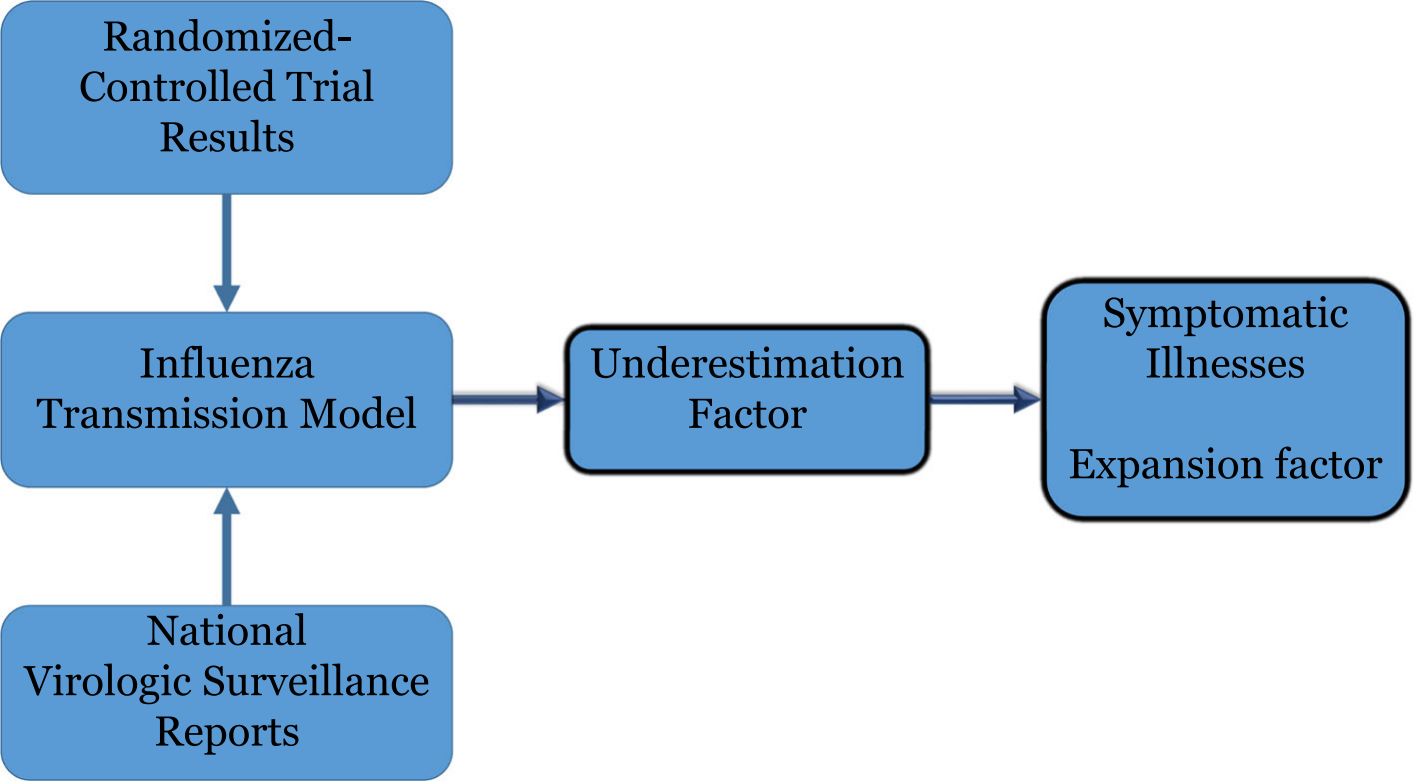
Methodology schematic. Data utilization and parameter estimation method. Quantifying the underestimation factor allows for the assessment of the expansion factor and symptomatic influenza illnesses

The present modelling study integrates multiple sources of data to generate estimates for the underestimation factor, disease transmission rate, expansion factor, and symptomatic influenza illnesses (Fig. 1). The laboratory-confirmed influenza cases during the 2011-2012 and 2012-2013 seasons are included in the final seasonal surveillance reports from FluWatch and FluView in Canada and the US, respectively [18, 19]. Additionally, we utilize the US and Canadian census profiles [20–23].

A recent RCT assessed the efficacy of a HD influenza vaccine compared to a SD influenza vaccine among 14,500 and 17,489 participants during the 2011-2012 and 2012-2013 seasons, respectively [17]. We utilize the laboratory-confirmed influenza counts among participants receiving SD and HD influenza vaccines in this RCT, which have been provided in the Supplementary Appendix of a prior publication [17]. The number of laboratory-confirmed influenza cases within the RCT are provided for three different case definitions [17]. In the present study, we utilize the laboratory-confirmed influenza cases associated with the least and most restrictive case definitions in the RCT. We utilize data associated with the least restrictive case definition, respiratory illness (RI), to provide estimates representing the true underestimation of symptomatic influenza illnesses. We utilize a more restrictive case definition, modified CDC-defined ILI, which is closer to the case definition used by surveillance systems in the US and Canada, to provide estimates representing underestimation of symptomatic influenza illnesses which were captured by virologic surveillance. RI was defined as the occurrence of one or more of the following: sneezing, nasal congestion or rhinorrhea, sore throat, cough, sputum production, wheezing, or difficulty breathing and modified CDC-defined ILI was defined as RI with cough or sore throat, concurrent with a temperature above 37.2 ^∘^C [17].

The SD influenza vaccine effectiveness and coverage over the 2011-2012 and 2012-2013 influenza seasons has been established in the US and Canada [24–27]. Also, the HD influenza vaccine coverage estimates have been made available in the US [28]. In Canada, the HD influenza vaccine was not yet licensed for use during 2011-2012 and 2012-2013 seasons.

### Mathematical modelling

We develop two compartmental models consisting of vaccinated and infected individuals for the 1) seniors aged 65+ who participated in the RCT and 2) the general population. Individuals are assumed to be vaccinated by the beginning of the influenza season, so there is no in-flow to the vaccinated compartment during the season. Since we study the dynamics of influenza within one season, we ignore the demographic dynamics (e.g. birth and death). As the size of the population for participants of the RCT is small relative to the total population, we ignore the influenza transmission from these participants to the general community; however, we assume the same transmission rate among the RCT participants. Also, we assume that the age groups 0-64 and 65+ are homogeneously mixed. In addition to homogeneous mixing by age group, we assume spatial homogeneity in transmission. We assign units of symptomatic influenza illness to the infected compartments of the following models for dimensional consistency with data.

#### Modelling influenza infections among RCT participants

We develop a compartmental model consisting of vaccinated and infected individuals for the seniors aged 65+ who participated in the RCT [17]. We make use of the following notation for model variables and parameters; the subscript ^′′^+^′′^ refers to compartments of individuals aged 65+ and subscript ^′′^−^′′^ refers to individuals aged 0-64. The vaccination status of the compartment will also appear in the subscript as SD (standard-dose) or HD (high-dose), if applicable. Lastly, let the superscript “O" denote individuals outside of the RCT and the superscript “C" denote participants within the RCT. With these assumptions and notations set, we formulate the following system of ordinary differential equations, where $V^{C}_{+,SD}$ represents RCT participants vaccinated given the standard-dose vaccine, $V^{C}_{+,HD}$ represents RCT participants vaccinated given the high-dose vaccine, $I^{C}_{+,HD}$ represents infected RCT participants with high-dose vaccine, and $I^{C}_{+,SD}$ represents the infected RCT participants given standard-dose vaccine. These model variables are described in Table 1. Submodel (1) is equipped with the following initial conditions to replicate the RCT conditions preceding each season: $V^{C}_{+,SD}(0) = V_{SD0}$, $V^{C}_{+,HD}(0) = V_{HD0}$, $I^{C}_{+,SD}(0) = 0$, $I^{C}_{+,HD}(0)=0$ [17].

1 $$ {}\begin{aligned} \dot{V}^{C}_{+,SD} &\,=\, - \epsilon_{+,SD} V^{C}_{+,SD} \beta \left(I^{C}_{+,SD}+I^{C}_{+,HD}+J\right) \\ \dot{V}^{C}_{+,HD} &\,=\, - \epsilon_{+,HD} V^{C}_{+,HD} \beta\left(I^{C}_{+,SD}+I^{C}_{+,HD}+J\right) \\\ \dot{I}^{C}_{+,SD} &\,=\, \epsilon_{+,SD} V^{C}_{+,SD} \beta\left(I^{C}_{+,SD}+I^{C}_{+,HD}+J\right) - \gamma I^{C}_{+,SD} \\ \dot{I}^{C}_{+,HD} &\,=\, \epsilon_{+,HD} V^{C}_{+,HD} \beta\left(I^{C}_{+,SD}+I^{C}_{+,HD}+J\right) - \gamma I^{C}_{+,HD}. \\ \end{aligned}  $$

**Table 1:** Model variables and descriptions for RCT influenza transmission submodel (1)

Model Variable	Description
$V^{C}_{+,SD}$	RCT participants vaccinated with SD
$V^{C}_{+,HD}$	RCT participants vaccinated with HD
$I^{C}_{+,SD}$	Infected RCT participants vaccinated with SD
$I^{C}_{+,HD}$	Infected RCT participants vaccinated with HD
*J*	Total, country-wide infected population

Parameters *ε*_+,*SD*_ and *ε*_+,*HD*_ denote the vaccine-modified susceptibilities corresponding to the standard-dose and high-dose influenza vaccine efficacy among seniors, respectively, *β* is the transmission rate and *γ* is the influenza recovery rate. Specifically, *β*=*b*/*N* where *b* is the daily effective contact rate and *N* is the total population size. The vaccine-modified susceptibilities *ε* represent a multiplier for the reduced infection rate of vaccinated individuals who are susceptible to influenza infection. Lastly, *J* represents the total infected individuals in the population from model (2), $J = I^{O}_{-} +I^{O}_{+}$.

#### Country-wide influenza transmission model

We subdivide the general community (i.e., the entire US or Canada) into two age groups: seniors aged 65+ and non-seniors aged 0-64, as well as vaccination status (SD or HD). The model variables and notations for model (2) are displayed in Table 2 and described in more detail below.

2 $$ {}\begin{aligned} \dot{S}^{O}_{-} &= - S^{O}_{-} \beta\left(I^{O}_{-}+I^{O}_{+}\right) \\ \dot{V}^{O}_{-,SD} &= -\epsilon_{-,SD} V^{O}_{-,SD}\beta \left(I^{O}_{-}+I^{O}_{+}\right) \\ \dot{I}^{O}_{-} &= S^{O}_{-} \beta (I^{O}_{-}+I^{O}_{+}) + \epsilon_{-,SD}V^{O}_{-,SD}\beta\left(I^{O}_{-}+I^{O}_{+}\right) -\gamma I^{O}_{-} \\\ \dot{S}^{O}_{+} &= - S^{O}_{+}\beta\left(I^{O}_{-}+I^{O}_{+}\right) \\ \dot{V}^{O}_{+,SD} &= -\epsilon_{+,SD} V^{O}_{+,SD} \beta\left(I^{O}_{-}+I^{O}_{+}\right) \\ \dot{V}^{O}_{+,HD} &= -\epsilon_{+,HD} V^{O}_{+,HD}\beta \left(I^{O}_{-}+I^{O}_{+}\right) \\ \dot{I}^{O}_{+} &= S^{O}_{+}\beta\left(I^{O}_{-}+I^{O}_{+}\right) + \epsilon_{+,HD} V^{O}_{+,HD} \beta\left(I^{O}_{-}+I^{O}_{+}\right) \\& \quad+ \epsilon_{+,SD} V^{O}_{+,SD} \beta\left(I^{O}_{-}+I^{O}_{+}\right) - \gamma I^{O}_{+} \end{aligned}  $$

**Table 2:** Model variables and descriptions for the general community (i.e., individuals outside of the RCT) influenza transmission model (2)

Model variable	Description
$S^{O}_{-}$	Population aged 0-64 susceptible to influenza infection
$S^{O}_{+}$	Population aged 65+ susceptible to influenza infection
$V^{O}_{-,SD}$	Vaccinated population aged 0-64 (Standard-dose)
$V^{O}_{+,SD}$	Vaccinated population aged 65+ (Standard-dose)
$V^{O}_{+,HD}$	Vaccinated population aged 65+ (High-dose)
$I^{O}_{-}$	Population aged 0-64 infected with influenza
$I^{O}_{+}$	Population aged 65+ infected with influenza

As identical with model (1), *β*=*b*/*N* where *b* is the daily effective contact rate and *N* is the total population size. Model (2) is equipped with the following initial conditions: the influenza season begins with the initial vaccinated population $V^{O}_{-,SD}(0) = \rho ^{O}_{-,SD} N_{-}$, $V^{O}_{+,SD}(0) = \rho ^{O}_{+,SD} N_{+}$, $V^{O}_{+,HD}(0) = \rho ^{O}_{+,HD} N_{+}$, where $\rho ^{O}_{-,SD}$, $\rho ^{O}_{+,SD}$, $\rho ^{O}_{+,HD}$ are the vaccine coverages for the population aged 0-64 and population aged 65 or over (either vaccinated with standard-dose or high-dose) in each year and country. Similarly, the initial susceptible populations are: $S^{O}_{-}(0)=\left (1-\rho ^{O}_{-,SD}\right)N_{-}$, $S^{O}_{+}(0)=\left (1-\rho ^{O}_{+,HD}-\rho ^{O}_{+,SD}\right)N_{+}$; there are no infections preceding the epidemic, so $I^{O}_{-}(0) = I^{O}_{+}(0)=0$. The values for these parameters embedded in the initial conditions are shown in Table 3 and estimated in “Parameter estimation” section.

**Table 3:** Parameters used for quantifying the influenza underestimation factor and transmission rate in the US and Canada for years 2011-2012 and 2012-2013, respectively

	

### Disease transmission and burden estimates

The approach for quantifying the underestimation factor and transmission rate utilizes the modelling framework developed in “Mathematical modelling” section. The mathematical details are presented in Appendix A. We assume that, since the RCT participants were actively monitored in the community, i.e. instructed to contact their study site if they had any respiratory symptoms and were, in addition, contacted weekly or bi-weekly by the site, that there was no underestimation of influenza infection within the RCT. The key idea is captured in the relationship *pJ*=*R*; a percent *p* (which denotes the underestimation factor) of symptomatic influenza cases *J* yields the laboratory-confirmed influenza cases *R*. We use this relationship *pJ*=*R*, the final epidemic size relationships for submodel (1) and model (2), and structure of the SVIR model to derive a tractable system of nonlinear equations. The representative equation derived from submodel (1) captures information from the RCT, while the representative equation derived from model (2) captures information from the general population. Specifically, we derive a nonlinear system of the form

3 $$ \begin{aligned}  f(\beta,p) = 0\\ g(\beta,p) =0, \end{aligned}  $$

where

4 $$ {}\begin{aligned} f(\beta, p) &\,=\, V^{C}_{+,SD}(\infty)\\&\quad \,-\, V^{C}_{+,SD}(0) e^{\frac{\epsilon_{+,SD} \beta}{\gamma}\! \left(\!V^{C}_{+,SD}(\infty) - V^{C}_{+,SD}(0) + V^{C}_{+,HD}(\infty) - V^{C}_{+,HD}(0) - p^{-1} \gamma \hat{R}\!\right)} \\ \noindent \text{and} & \\ g(\beta,p) &= e^{-\left(\beta p^{-1}\hat{R}\right) }\left(S^{O}_{-}(0)+S^{O}_{+}(0)\right) +V^{O}_{-,SD}(0)e^{-\left(\beta \epsilon_{-,SD} p^{-1}\hat{R}\right)} \\ &+V^{O}_{+,SD}(0)\left(e^{-\left(\beta \epsilon_{+,SD} p^{-1}\hat{R}\right) }\right)+ V^{O}_{+,HD}(0) e^{-\left(\beta \epsilon_{+,HD} p^{-1}\hat{R}\right)} \\ &-\left(S^{O}_{-}(0)+ S^{O}_{+}(0)+ V^{O}_{-,SD}(0)+V^{O}_{+,SD}(0)+ V^{O}_{+,HD}(0)\right) +\gamma p^{-1}\hat{R}, \end{aligned}  $$

and $\hat {R}$ is the laboratory-confirmed cases in an influenza season captured in national virological surveillance. To solve this system of nonlinear equations (4) for *p* and *β*, we use Matlab’s *fsolve* function in Matlab R2016a.

#### Expansion factor and symptomatic illnesses

The expansion factor, *E*, is defined in this study as the number of symptomatic influenza illnesses per laboratory-confirmed infection. In terms of the underestimation factor, the expansion factor is its multiplicative inverse. Hence, we compute the expansion factor by finding the multiplicative inverse of *p*, that is *E*=*p*^−1^.

The number of estimated symptomatic influenza illnesses is $E \hat {R}$ where $\hat {R}$ is the total laboratory-confirmed influenza cases from national surveillance during an influenza season.

The basic reproduction number is the average number of secondary cases produced by one infected individual introduced into a population of susceptible individuals. We determine the basic reproduction number of model (2) using the next generation method [35].

### Parameter estimation

We determine estimates for parameters associated with submodel (1) and model (2) utilizing data in “Data sources” section. We provide an outline of the methods in the main text and more complete calculations and explanations are included in Appendix B.

*Estimating vaccine-modified susceptibilityε*_−,*SD*_, *ε*_+,*SD*_*andε*_+,*HD*_: Recall the vaccine-modified susceptibility *ε* captures the protection added from vaccination among vaccinated individuals against influenza infection. Here we outline the method for estimating parameter values *ε*_+,*SD*_,*ε*_+,*HD*_ and *ε*_−,*SD*_ for influenza seasons 2011-2012 and 2012-2013. This process integrates model analysis and prior vaccine effectiveness (VE) studies. We make use of a relationship between vaccine-modified susceptibility and VE [36].

To infer *ε*_−,*SD*_ and *ε*_+,*SD*_ we use prior estimates of vaccine effectiveness (VE) against influenza. Specifically, we relate these VE estimates to vaccine-modified susceptibility *ε* with the relationship *ε*=1−VE [36]. Remaining is to find *ε*_+,*HD*_, which we use *ε*_+,*SD*_ and the ratio *ε*_+,*HD*_/*ε*_+,*SD*_. We use submodel (1) to determine the ratio *ε*_+,*HD*_/*ε*_+,*SD*_. Specifically, we find *ε*_+,*HD*_/*ε*_+,*SD*_ by dividing the first two equations in submodel (1), which yields $\frac {\dot {V}^{C}_{+,HD}}{\dot {V}^{C}_{+,SD}} = \frac {\epsilon _{+,HD} V^{C}_{+,HD}}{\epsilon _{+,SD} V^{C}_{+,SD}}$. Finally, we use separation of variables to find the ratio in terms of known RCT outcomes [17]:

5 $$ \frac{\epsilon_{+,HD}}{\epsilon_{+,SD}} = \frac{\log\left(V^{C}_{+,HD}(\infty)/V^{C}_{+,HD}(0)\right)}{\log\left(V^{C}_{+,SD}(\infty)/V^{C}_{+,SD}(0)\right)}.  $$

The quantities $V^{C}_{+,SD}(\infty)$ and $V^{C}_{+,HD}(\infty)$ are the limit values of the state variables $V^{C}_{+,SD}$ and $V^{C}_{+,HD}$, respectively. Finally, we use estimates of now known quantities; *ε*_+,*SD*_ from VE studies and the ratio $\frac {\epsilon _{+,HD}}{\epsilon _{+,SD}}$ from the RCT to estimate *ε*_+,*HD*_ [17].

*Initial susceptible and vaccinated populations:* To inform the initial conditions for model (2) in the years 2011-2012 and 2012-2013, we use population sizes given by the US and Canadian census programs [20–23]. The population size and the estimated vaccine coverage in each country then gives us the susceptible and vaccinated population initial conditions. The values and descriptions of these parameters embedded in the initial conditions; $N_{-}, N_{+}, \rho ^{O}_{-,SD}, \rho ^{O}_{+,SD}, \text {and} \rho ^{O}_{+,HD}$, are listed in Table 3. Similarly, the initial conditions for submodel (1) are given in Table 3.

*Influenza recovery rateγ*: We inform the recovery rate *γ* using the infectious period of influenza, which has been estimated to be 3.8 days with a 95% confidence interval (CI) of 3.1 - 4.6 days [34]. The recovery rate *γ* is then the inverse of the mean sojourn time in the infectious compartment; hence, we consider $\gamma = \frac {1}{3.8} \text { day}^{-1}$ as a baseline. We utilize the bounds on the CI of the estimated infectious period for sensitivity analysis and vary *γ* from $\frac {1}{4.6}$ to $\frac {1}{3.1} \text { day}^{-1}$.

We utilize the laboratory-confirmed influenza counts reported in the RCT according to two case definitions to inform $V^{C}_{+,HD}(\infty)$ and $V^{C}_{+,SD}(\infty)$. We use 1) laboratory-confirmed cases associated with modified CDC-defined ILI and 2) laboratory-confirmed cases associated with respiratory illness (RI) [17] (Table 3). Results generated for each case definition has an interpretation and is left for the Discussion.

### Well-posedness

To ensure that the parameter estimation process for obtaining *p* and *β* will yield biologically relevant results, we study the well-posedness of the inverse problem outlined in “Disease transmission and burden estimates” section. The solution *p* and *β* to system (4) is unique and positive, i.e. the problem is well-posed. From *f*(*β*,*p*)=0, we note that *β* is related to *p* by the following equation

$$\begin{aligned} \beta &= - \ln\frac{V^{C}_{+,SD}(\infty)}{V^{C}_{+,SD}(0)} \\&\quad \cdot \frac{\gamma}{\epsilon_{+,SD} V^{C}_{+,SD}(0)\left(1\,-\,\frac{V^{C}_{+,SD}(\infty)}{V^{C}_{+,SD}(0)}\right)\,+\, V^{C}_{+,HD}(0)\left(1-\frac{V^{C}_{+,HD}(\infty)}{V^{C}_{+,HD}(0)}\right) +p^{-1} \gamma \hat{R})}. \end{aligned} $$

Substituting this expression for *β* into *g*(*β*,*p*) yields the following equation of *p*

6 $$ \begin{aligned} &\left(S^{O}_{-}(0)+S^{O}_{+}(0)+ V^{O}_{-,SD}(0)+V^{O}_{+,SD}(0)+ V^{O}_{+,HD}(0)\right)-\\ &\left(S^{O}_{-}(0)+S^{O}_{+}(0)\right)\left(\frac{V^{C}_{+,SD}(\infty)}{V^{C}_{+,SD}(0)}\right)^{U(p)}-V^{O}_{-,SD}(0)\left(\frac{V^{C}_{+,SD}(\infty)}{V^{C}_{+,SD}(0)}\right)^{U(p)}\\ &-V^{O}_{+,SD}(0)\left(\frac{V^{C}_{+,SD}(\infty)}{V^{C}_{+,SD}(0)}\right)^{\epsilon_{+,SD} U(p)}-V^{O}_{+,HD}(0)\left(\frac{V^{C}_{+,SD}(\infty)}{V^{C}_{+,SD}(0)}\right)^{\epsilon_{+,HD} U(p)}\\ &=\gamma p^{-1}\hat{R} \end{aligned}  $$

where

$$\begin{aligned} &U(p)=\\ &\frac{\gamma\hat{R}p^{-1}}{\epsilon_{+,SD}\left(\left(V^{C}_{+,SD}(0)\left(1-\frac{V^{C}_{+,SD}(\infty)}{V^{C}_{+,SD}(0)}\right)+V^{C}_{+,HD}(0)\left(1-\frac{V^{C}_{+,HD}(\infty)}{V^{C}_{+,HD}(0)}\right)+p^{-1}\gamma\hat{R}\right.\right)}. \end{aligned} $$

Let *Φ*(*p*) denote the left hand side of equation (6). Note that *Φ* is a monotone-decreasing function of *p* with *Φ*(0)>0 and ${\lim }_{p\to \infty } \Phi (p)=0$. On the other hand, the right hand side of the equation is a monotone-decreasing function of *p* with ${\lim }_{p\to 0} \gamma \hat {R}p^{-1}=\infty $ and ${\lim }_{p\to \infty } \gamma \hat {R}p^{-1}=0$. Therefore, there exists a unique solution *p*∈(0,1] if $\Phi (1)>\gamma \hat {R}$.

From the parameter sets estimated in “Parameter estimation” section, $\Phi (1) > \gamma \hat {R}$, hence we have a unique solution of System (3) corresponding to each context-specific parameter set. In other words, according to this specific US/Canadian demographic information, vaccine-specific parameters, influenza surveillance reports, and RCT study results, there is a single underestimation factors *p* (which we ensure is logically between 0 and 1) and transmission rate *β* which satisfy System (3) [17–19].

### Sensitivity analysis

To quantify uncertainty in the underestimation factor, expansion factor, number of symptomatic influenza illnesses, and basic reproduction number, we utilize variability in parameter estimates in Table 3. Note that model parameter estimates appear as point values with the exception of the recovery rate *γ* (Table 3). We solve system (4) according to each case definition (Modified-CDC and RI), country (US and Canada) and study year (2011-2012 and 2012-2013) with the mean value of *γ*, lower 95% confidence bound and upper 95% confidence bound on *γ*. The estimated value of *p* and *β* corresponding to the mean value of *γ* represent baseline results. The ranges of *p* and *β* obtained using the 95% confidence bounds of *γ* represent their sensitivity to an estimated 95% CI of the infectious period of influenza. We obtain baseline estimates of the expansion factor, number of symptomatic influenza illnesses and the basic reproduction number using baseline *p* and *β* estimates and methods in “Expansion factor and symptomatic illnesses” section. To retrieve an interval based on variation of recovery rate *γ*, we propagate the variability in *p* and *β* to each of the epidemiological parameters using methods in “Expansion factor and symptomatic illnesses” section.

### Sensitivity to underestimation in the RCT

We have assumed perfect reporting and ascertainment of influenza virus infection within the RCT. Participants were instructed to contact their study site if they had any respiratory symptoms [17]. In addition, participants were contacted by a call center twice weekly (between the beginning of January and the end of February) or weekly until the end of illness surveillance (April 30 each year) [17]. In light of these frequent participant follow-ups in the RCT, we expect this assumption to hold. However, we consider the possibility of underestimation occurring within the RCT to be exhaustive in our analysis [17]. For details regarding the sensitivity of *p* and *β* to underestimation in the RCT, see Appendix C.

## Results

### Disease transmission and burden estimates

**Fig. 2:**
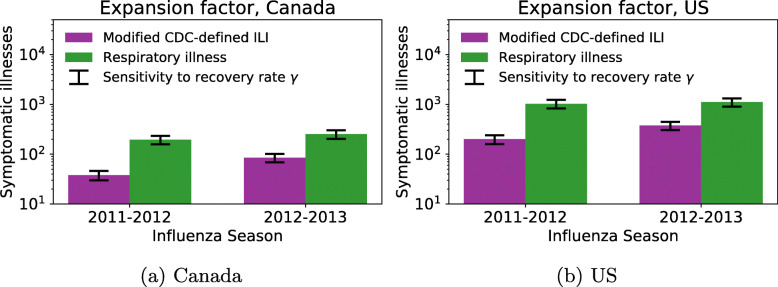
Expansion factor in the US and Canada during the 2011-2012 and 2012-2013 seasons according to case definition. See section “Discussion” for comparisons with prior estimates and Tables 4 and 5 for the estimate values and ranges

The estimates for Canada are displayed in Table 4. Using the laboratory-confirmed influenza counts associated with modified CDC-defined ILI within the RCT, we quantified the underestimation factor *p*=2.6*%*(2.1−3.2*%*) and *p*=1.2*%*(0.98−1.2*%*) in the 2011-2012 and 2012-2013 influenza seasons, respectively. These underestimation factors correspond to expansion factors of *E* = 37.9 (31−46.8) and *E*=84.8(69.1−102.6) in 2011-2012 and 2012-2013 influenza seasons, respectively (Fig. 2a). Equipped with expansion factors in each year, we estimated symptomatic influenza illnesses to be 390,000 (320,000−480,000) and 2.3 million (1.8−2.7 million) in 2011-2012 and 2012-2013 influenza seasons, respectively. Also in Canada, using the laboratory-confirmed influenza counts associated with RI within the RCT, we quantified the underestimation factor *p* = 0.51% (0.42−0.63*%*) and *p*=0.4*%*(0.33−0.49*%*) in the 2011-2012 and 2012-2013 influenza seasons, respectively. These underestimation factors correspond to expansion factors of *E* = 200.1 (163.4−243.9) and *E* = 376.1 (306.7−455.1) in 2011-2012 and 2012-2013 influenza seasons, respectively. Equipped with expansion factors in each year, we estimated symptomatic influenza illnesses to be 2 million (1.6−2.4 million) and 6.7 million (5.5−8.2 million) in 2011-2012 and 2012-2013 influenza seasons, respectively.

**Table 4:** Summary of estimates in Canada during 2011-2012 and 2012-2013 influenza seasons. Values reported as estimated baseline value and range from variation of recovery rate *γ* (see section “Sensitivity analysis” for the details of sensitivity analysis)

Seasonal influenza in Canada: 2011-2012 and 2012-2013 influenza seasons		
Laboratory-confirmed infections*	10,271	26,671
Estimates generated from modified CDC-defined influenza in RCT		
Season	2011-12	2012-13
Percent of influenza cases captured in surveillance	2.6*%*(2.1−3.2*%*)	1.2*%*(0.98−1.2*%*)
Expansion factor	37.9 (31.0−46.8)	84.8 (69.1−102.6)
Estimated symptomatic influenza illnesses	3.9×10^5^(3.2×10^5^−4.8×10^5^)	2.3×10^6^(1.8×10^6^−2.7×10^6^)
Basic reproduction number	1.22 (1.22−1.22)	1.19 (1.19−1.19)
Estimates generated from respiratory illness associated influenza in RCT		
Season	2011-12	2012-13
Percent of influenza cases captured in surveillance	0.51*%*(0.42−0.63*%*)	0.4*%*(0.33−0.49*%*)
Expansion factor	195.1 (159.3−236.1)	252.9 (206.3−306.1)
Estimated symptomatic influenza illnesses	2.0×10^6^(1.6×10^6^−2.4×10^6^)	6.7×10^6^(5.5×10^6^−8.2×10^6^)
Basic reproduction number	1.22 (1.22−1.22)	1.19 (1.19−1.19)

^*^indicates infections recorded in Public Health Agency of Canada’s final seasonal FluWatch report [19]

**Table 5:** Summary of estimates in the US during 2011-2012 and 2012-2013 influenza seasons. Values reported as estimated baseline value and range from variation of recovery rate *γ* (see section “Sensitivity analysis” for the details of sensitivity analysis)

Seasonal influenza in the US: 2011-2012 and 2012-2013 influenza seasons		
Laboratory-confirmed infections*	27,012	75,772
Estimates generated from Modified CDC-defined ILI in RCT		
Season	2011-12	2012-13
Percent of influenza cases captured in surveillance	0.5*%*(0.41−0.61*%*)	0.27*%*(0.2−0.33*%*)
Expansion factor	200.1 (163.4−243.9)	376.1 (306.7−455.1)
Estimated symptomatic influenza illnesses	5.4×10^6^(4.4×10^6^−6.9×10^6^)	2.9×10^7^(2.3×10^7^−3.4×10^7^)
Basic reproduction number	1.19 (1.19−1.19)	1.16 (1.16−1.16)
Estimates generated from respiratory illness associated influenza in RCT		
Season	2011-12	2012-13
Percent of influenza cases captured in surveillance	0.1*%*(0.08−0.12*%*)	0.09*%*(0.07−0.11*%*)
Expansion factor	1030.1 (840.5−1247.0)	1111.4 (906.6−1345.3)
Estimated symptomatic influenza illnesses	2.8×10^7^(2.3×10^7^−3.4×10^7^)	8.4×10^7^(6.9×10^7^−1.02×10^8^)
Basic reproduction number	1.20 (1.20−1.20)	1.19 (1.19−1.19)
US CDC estimated symptomatic influenza illnesses	9.3×10^6^	3.4×10^7^

^*^Indicates infections recorded by the US Center for Disease and Control’s FluView reports [18]

The estimates for the US are displayed in Table 5. Using the laboratory-confirmed influenza associated with modified CDC-defined ILI within the RCT, we estimated *p*=0.5*%*(0.41−0.61*%*) and *p*=0.27*%*(0.2−0.33*%*) in the 2011-2012 and 2012-2013 seasons, respectively. These underestimation factors correspond to expansion factors of *E* = 200.1 (163.4−243.9) and *E* = 376.1 (306.7−455.1) in 2011-2012 and 2012-2013 influenza seasons, respectively (Fig. 2b). Equipped with expansion factors in each year, we estimated symptomatic influenza illnesses to be 5.4 million (4.4−6.9 million) and 29 million (23−34 million) in 2011-2012 and 2012-2013 influenza seasons, respectively. Also in the US, using the laboratory-confirmed influenza counts associated with RI within the RCT, we estimated *p*=0.1*%*(0.08−0.12*%*) and *p*=0.09*%*(0.07−0.11*%*) in the 2011-2012 and 2012-2013 seasons, respectively. These underestimation factors correspond to expansion factors of *E* = 1030.1 (840.5−1247) and *E* = 1111.4 (906.6−1345.3) in 2011-2012 and 2012-2013 influenza seasons, respectively. Equipped with expansion factors in each year, we estimated symptomatic influenza illnesses to be 28 million (23−34 million) and 84 million (69−102 million) in 2011-2012 and 2012-2013 influenza seasons, respectively.

We estimated the disease transmission rate *β* range to between 0.25 and 0.39 in each season and country, corresponding to basic reproduction numbers *R*_0_ ranging between 1.19 and 1.22 (Tables 4 and 5, RI case definition).

### Sensitivity to underestimation in RCT

Recall we have assumed perfect reporting and ascertainment of influenza virus infection among the RCT participants and proposed to revisit this assumption with a sensitivity analysis. We have conducted a sensitivity analysis and present the details in Appendix C. Overall, the sensitivity analysis suggests that the results in “Results” section are robust to underestimation within the RCT [17]. We find that our estimates for *β* are weakly dependent on underestimation within the RCT [17]. Further, the captured fraction of influenza infections by virologic surveillance *p* is also robust to underestimation within the RCT [17], maintaining an order of magnitude while varying RCT underestimation over its full range. See Appendix C for a full presentation of this analysis.

## Discussion

This study develops and illustrates a method utilizing a parameter estimation technique based on a mechanistic model and data synthesis to quantify the underestimation factor associated with season influenza and the number of symptomatic illnesses. These estimates take into account mechanistic detail of influenza transmission, vaccine effectiveness, relative vaccine efficacy of SD to HD from the RCT, and vaccine coverage. While this method does utilize RCT outcomes, the remaining data required to generate the estimates become publicly available by the end of the influenza season. This method can be utilized for epidemiological parameter estimation for infectious diseases, provided there is an appropriate form of active surveillance (e.g., clinical trial) data available. A coupled system of differential equations for the actively monitored population and general community can be developed to integrate surveillance epidemiological data to quantify key population-level parameters, including the underestimation factor.

Our estimates generated from the laboratory-confirmed influenza associated with modified CDC-defined ILI case definition are representative of the underestimation of cases which could be captured by the surveillance system (Tables 4 and 5, modified CDC-defined ILI case definition). In this light, our analysis indicates that the surveillance system in Canada captured 2.6*%*(2.1−3.2*%*) and 1.2*%*(0.98−1.2*%*) of symptomatic cases closely associated with modified CDC-defined ILI in 2011-2012 and 2012-2013 influenza seasons, respectively. In the US, the virologic surveillance system was estimated to capture 0.1*%*(0.08−0.12*%*) and 0.09*%*(0.07−0.11*%*) of symptomatic influenza cases closely associated with modified CDC-defined ILI in 2011-2012 and 2012-2013 influenza seasons, respectively. In each country, the percentage of captured cases decreases with an increase in laboratory-confirmed cases from passive surveillance, which may be due to laboratory testing capacity or changing testing practices (Tables 4 and 5, modified CDC-defined ILI).

**Fig. 3:**
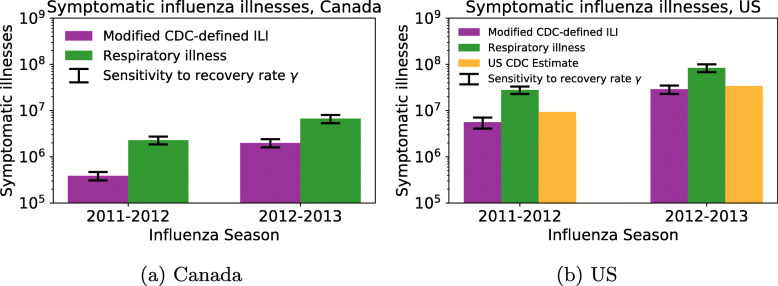
Estimated symptomatic influenza illnesses in the Canada and the US during the 2011-2012 and 2012-2013 seasons, by case definition. **b** In the US, we compare with US CDC’s estimates of symptomatic influenza illnesses [14]. For data values and ranges see Tables 4 and 5

The underestimation factors generated from laboratory-confirmed influenza associated with RI, i.e., a broader range of symptoms, provide an underestimation factor more closely representing the true symptomatic influenza underestimation factor and are apt to assess the true number of influenza illnesses (Tables 4 and 5, respiratory illness case definition). When considering a range of symptomatic influenza illness from the estimates using data specified by these two case definitions, we provide symptomatic influenza illness estimates which are consistent with US CDC’s in 2011-2012 and 2012-2013 (Fig. 3b). The US CDC estimate in 2012-2013 is near the low end of the range we estimated, which may be due to difference in methodology. In particular, this difference may be due to the nonlinear (exponential) relationship between the underestimation factor and laboratory-confirmed illnesses in surveillance data. An explanation to support this nonlinear relationship and our results could be that the increased number of influenza illnesses results in limited availability of laboratory tests, therefore a fewer proportion of cases were captured in virological surveillance.

We offer two sources of comparison from studies of the 2009 influenza pandemic. In an expert opinion from the 2009 influenza pandemic, the number of expected influenza infections per laboratory-confirmed infection ranged from 10 to 500 among experts [37]. For Canada, our estimates of the expansion factor lie in this range of 10 to 500 (Table 4). For the US, we estimate expansion factors greater than 500 using respiratory illness case definition (Table 5). Also, estimates of underestimation factors were between 0.1% and 0.7% during the 2009 influenza pandemic in Mexico [16]. Our estimates for the true underestimation in Canada and the US lie in this range (Table 4 and 5, respiratory illness case definition). In both cases, investigations of the 2009 pandemic influenza in Canada and Mexico align well with Canada; however, according to our analysis the underestimation in laboratory-confirmed surveillance in the US is more substantial. This is likely due to differences in surveillance systems, laboratory-testing protocols, or health-seeking behavior. We also note that epidemiological characteristics differ from pandemics to seasonal influenza and the level of awareness may have impacted health seeking behavior and diagnosis practices.

The basic reproduction number ranged from 1.19 - 1.22 for estimates associated with RI case definition, which more closely represent the true *R*_0_. While these basic reproduction numbers align tightly in the US and Canada, our estimates for *R*_0_ are higher in Canada in all influenza seasons (Tables 4 and 5). A recent systematic review found an interquartile range of reproduction numbers for seasonal influenza of 1.19–1.37, with median value 1.27 [38]. Our estimates for *R*_0_ are in line with typical findings representative of seasonal influenza [38].

We estimate the symptomatic influenza illnesses in the US and Canada; however, the number of asymptomatic influenza illnesses has not been assessed in this work. A recent review of estimates for the fraction of asymptomatic infections vary widely; however, values from 20%-50% are typical [39]. These estimates indicate that the number of asymptomatic influenza illnesses may be substantial. Even so, based on our scan of the literature, there is no clear consensus on the contribution of asymptomatic individuals to influenza transmission. Future studies may attempt to estimate the total number influenza illnesses, symptomatic and asymptomatic, to provide a more accurate assessment of influenza illnesses; influenza underestimation; and the force of infection. As a result of this simplification, we may underestimate *R*_0_ and hence the force of infection.

There are several limitations in the current study. There are differences in symptomatic attack rates, disease presentation, and health-seeking behaviour between age groups. As a result, the symptomatic reporting fraction likely varies from age group to age group. Future study may be extended to address these age group disparities by quantifying the underestimation and expansion factors for each age group. Another limitation to this study is the assumed homogeneous mixing between age groups; however, this may also be addressed in a future study. In fact, the RCT data may allow the contact preference between age groups (i.e. the contact rate between age groups 0-64 and 65+, and vice-versa) to be estimated. In this light, the methods presented herein may be used as a method to validate empirical social contact data obtained from surveys or existing contact mixing patterns. In addition to age-specific heterogeneity, there may be spatial heterogeneity in contact mixing, surveillance systems, and social behavior which impacts case ascertainment and reporting rates. In this light, it may be advantageous to conduct analysis at more granular scales (e.g. city, state, or provincial levels) to more accurately capture transmission and underestimation at these sub-national levels. The RCT was conducted in the US and Canada, and we use aggregate case counts and RCT participant counts from US and Canada. In other words, the RCT is assumed to take place in the US or Canada. The underestimation factor could also be separated into several multipliers, as in prior works [13]. An improvement could be made to account for laboratory test accuracy; laboratory-confirmed cases from the RCT and surveillance reports can be preprocessed for the method to generate more representative estimates. Overall, this work lays a functional framework to expand as the research questions at hand require.

## Conclusions

We develop a method for quantifying the underestimation factor and disease transmission rate by integrating several data sources including RCT outcomes. We use our method to assess surveillance system capacity, number of symptomatic influenza illnesses, and *R*_0_ in the US and Canada during 2011-2012 and 2012-2013 seasons. The utilization of outcomes from RCTs (in this case a comparative vaccine RCT) may allow for the extraction of additional information characterizing an epidemic which may not be possible by limiting data usage to national influenza surveillance reports. This work illustrates the point that monitoring a representative group within a population may aid in effectively modelling the transmission of infectious diseases such as influenza. In general, the utilization of available surveillance data may increase the capabilities of disease models and broaden their power to draw inferences. A formal structural and practical identifiability analysis should be carried out to rigorously address these details of parameter identification.

It is key to provide more accurate methods to estimate the annual incidence of influenza to guide evidence-based immunization policy-making. In this light, it is vital to develop more accurate mathematical models of influenza transmission, and to accurately evaluate the impact of influenza vaccination. These methods may contribute to and enable the design of optimal vaccination programs that best reduce the annual incidence of seasonal influenza as well as associated hospitalizations, medical visits and deaths.

## Appendix A: Mathematical details for estimating *p* and *β*.

Here we present the details for estimating *p* and *β*. We also introduce the following notation for convenience and readability; let a hat ($\hat {}$) over a variable denote integration over all nonnegative time. For example, $\hat {I} = \int _{0}^{\infty }I(s)ds$.

Integrating the first equation of submodel (1) we have

7 $$  \ln\frac{V^{C}_{+,SD}(\infty)}{V^{C}_{+,SD}(0)} = -\epsilon_{+,SD} \beta (\hat{I}^{C}_{+,SD}+ \hat{I}^{C}_{+,HD} + \hat{J}).  $$

Similarly, taking the second equation from submodel (1), we have:

8 $$  \ln\frac{V^{C}_{+,HD}(\infty)}{V^{C}_{+,HD}(0)} = -\epsilon_{+,HD} \beta (\hat{I}^{C}_{+,SD}+ \hat{I}^{C}_{+,HD} + \hat{J}).  $$

For finding $\hat {I}^{C}_{+,SD}$, $\hat {I}^{C}_{+,HD}$, adding the first and third equations, & second and fourth of submodel (1) followed by integrating both sides give us:

$$\hat{I}^{C}_{+,SD} = -\frac{V^{C}_{+,SD}(\infty) - V^{C}_{+,SD}(0) }{\gamma}, $$

$$\hat{I}^{C}_{+,HD} = -\frac{V^{C}_{+,HD}(\infty) - V^{C}_{+,HD}(0) }{\gamma}, $$

provided that $I_{+,SD}^{C}(0) = I_{+,SD}^{C}(\infty) = I_{+,HD}^{C}(0) = I_{+,HD}^{C}(\infty) =0.$ This, along with *pJ*=*R* (where $J = I^{O}_{-}+I^{O}_{+}$), from Eqs. 7 and (8) we have:

$${}\begin{aligned} &\frac{V^{C}_{+,SD}(\infty)}{V^{C}_{+,SD}(0)}\\ &\quad \!= \!e^{\frac{\epsilon_{+,SD} \beta}{\gamma} (V^{C}_{+,SD}(\infty) - V^{C}_{+,SD}(0) + V^{C}_{+,HD}(\infty) - V^{C}_{+,HD}(0) - p^{-1} \gamma \hat{R})} \end{aligned} $$

and

$${}\begin{aligned} &\frac{V^{C}_{+,HD}(\infty)}{V^{C}_{+,HD}(0)}\\ &\quad= e^{\frac{\epsilon_{+,HD} \beta}{\gamma} (V^{C}_{+,SD}(\infty) - V^{C}_{+,SD}(0) + V^{C}_{+,HD}(\infty) - V^{C}_{+,HD}(0) - p^{-1} \gamma \hat{R})}. \end{aligned} $$

Thus, we define the following functions:

9 $$ \begin{aligned} f(\beta, p) &= V^{C}_{+,SD}(\infty)\\ &\quad-\! V^{C}_{+,SD}(0) e^{\frac{\epsilon_{+,SD} \beta}{\gamma}\! \left(\!V^{C}_{+,SD}(\infty) - V^{C}_{+,SD}(0) + V^{C}_{+,HD}(\infty) - V^{C}_{+,HD}(0) - p^{-1} \gamma, \hat{R}\!\right)} \end{aligned}  $$

10 $$ \begin{aligned} h(\beta, p) &= V^{C}_{+,HD}(\infty)\\ &- V^{C}_{+,HD}(0) e^{\frac{\epsilon_{+,HD} \beta}{\gamma} (V^{C}_{+,SD}(\infty) - V^{C}_{+,SD}(0) + V^{C}_{+,HD}(\infty) - V^{C}_{+,HD}(0) - p^{-1} \gamma \hat{R})}. \end{aligned}  $$

Now, from model (2) we have the following five equations:

$$\begin{aligned} {}\frac{\dot{S^{O}_{-}}}{S^{O}_{-}}&=-\beta J \mathrm{,}\quad \frac{\dot{S}^{O}_{+}}{S^{O}_{+}}=-\beta J \mathrm{,}\quad \frac{\dot{V}^{O}_{-,SD}}{V^{O}_{-,SD}}=- \epsilon_{-,SD} \beta J \mathrm{,}\\ \frac{\dot{V}^{O}_{+,SD}}{V^{O}_{+,SD}}&=- \epsilon_{+,SD} \beta J \mathrm{,}\quad \frac{\dot{V}^{O}_{+,HD}}{V^{O}_{+,HD}}=-\epsilon_{+,HD}\beta J. \end{aligned} $$

Recall *pJ*=*R*, where *p* is the underestimation factor. Integrating the equations above yields the respective final size relations:

$${\kern20pt}\begin{aligned} S^{O}_{-}(\infty)&=S^{O}_{-}(0) e^{-(\beta p^{-1}\hat{R}) }\mathrm{,}\\ S^{O}_{+}(\infty)&=S^{O}_{+}(0) e^{-(\beta p^{-1}\hat{R})},\\ V^{O}_{-,SD}(\infty)&=V^{O}_{-,SD}(0) e^{-(\beta \epsilon_{-,SD} p^{-1}\hat{R})} \mathrm{,}\\ V^{O}_{+,SD}(\infty)&=V^{O}_{+,SD}(0) e^{-(\beta \epsilon_{+,SD} p^{-1}\hat{R})} \mathrm{,}\\ V^{O}_{+,HD}(\infty)&=V^{O}_{+,HD}(0) e^{-(\beta \epsilon_{+,HD} p^{-1}\hat{R})}. \end{aligned} $$

Further, by summing all the equations of model (2), we obtain the following equality:

$$\dot{S}^{O}_{-}+\dot{S^{O}_{+}}+\dot{V}^{O}_{-,SD}+\dot{I}^{O}_{+}+\dot{V}^{O}_{+,SD}+\dot{V}^{O}_{+,HD}+\dot{I}^{O}_{-}=-\gamma J. $$

Integrating both sides of the above equations with the assumption that there are no infections at *t*_0_ and *t*_*∞*_ gives:

11 $$\begin{array}{@{}rcl@{}} &&S^{O}_{-}(\infty) -S^{O}_{-}(0)+S^{O}_{+}(\infty)-S^{O}_{+}(0)\\&&+(V^{O}_{-,SD}(\infty) -V^{O}_{-,SD}(0))  \\ &&+ \left(V^{O}_{+,SD}(\infty) -V^{O}_{+,SD}(0)\right) + \left(V^{O}_{+,HD}(\infty) -V^{O}_{+,HD}(0)\right) \\ &&= -\gamma p^{-1}\hat{R}. \end{array} $$

Substituting values of final sizes from above relations and $p\hat {J} = \hat {R}$ into (11):

$$\begin{array}{*{20}l} {}&S^{O}_{-}(0)e^{-(\beta p^{-1}\hat{R})} -S^{O}_{-}(0) +\left(V^{O}_{-,SD}(0) e^{-(\beta \epsilon_{-,SD} p^{-1}\hat{R}) } \right.\\ &-\left.V^{O}_{-,SD}(0)) +(V^{O}_{+,SD}(0)e^{-(\beta \epsilon_{+,SD} p^{-1}\hat{R})} -V^{O}_{+,SD}(0)\right)\\ &+\left(V^{O}_{+,HD}(0) e^{-(\beta \epsilon_{+,HD} p^{-1}\hat{R})} -V^{O}_{+,HD}(0)\right) = -\gamma p^{-1}\hat{R} \end{array} $$

Thus we define:

12 $$ \begin{aligned} g(\beta,p)&= e^{-(\beta p^{-1}\hat{R}) }\left(S^{O}_{-}(0)+S^{O}_{+}(0)\right) +V^{O}_{-,SD}(0) e^{-(\beta \epsilon_{-,SD} p^{-1}\hat{R})} \\ &\quad+V^{O}_{+,SD}(0)\left(e^{-(\beta \epsilon_{+,SD} p^{-1}\hat{R}) }\right)+ V^{O}_{+,HD}(0) \left(e^{-\left(\beta \epsilon_{+,HD} p^{-1}\hat{R}\right)}\right) \\ &\quad-\left(S^{O}_{-}(0)+S^{O}_{+}(0)+ V^{O}_{-,SD}(0)+V^{O}_{+,SD}(0)+ V^{O}_{+,HD}(0)\right) +\gamma p^{-1}\hat{R}. \end{aligned}  $$

With this definition, we obtain the following system of equations, which *β* and *p* must satisfy:

13 $$ \begin{aligned}  f(\beta,p) = 0,\\ g(\beta,p) =0. \end{aligned}  $$

Suppose that *β* and *p* satisfy *f*(*β*,*p*)=0, then we have:

$$\begin{aligned} \beta &= - \ln\frac{V^{C}_{+,SD}(\infty)}{V^{C}_{+,SD}(0)}\\ &\quad\cdot \frac{\gamma}{\epsilon_{+,SD} (V^{C}_{+,SD}(\infty) - V^{C}_{+,SD}(0) + V^{C}_{+,HD}(\infty) - V^{C}_{+,HD}(0) - p^{-1} \gamma \hat{R})} \end{aligned} $$

and that *β* and *p* also satisfy *h*(*β*,*p*)=0, that is

$$\begin{aligned} \beta &= - \ln\frac{V^{C}_{+,HD}(\infty)}{V^{C}_{+,HD}(0)}\\ &\cdot \frac{\gamma}{\epsilon_{+,HD} (V^{C}_{+,SD}(\infty) - V^{C}_{+,SD}(0) + V^{C}_{+,HD}(\infty) - V^{C}_{+,HD}(0) - p^{-1} \gamma \hat{R})}. \end{aligned} $$

Note that for both of these equalities to hold, it is required that

14 $$  \frac{1}{\epsilon_{+,SD}}\ln\frac{V^{C}_{+,SD}(\infty)}{V^{C}_{+,SD}(0)} = \frac{1}{ \epsilon_{+,HD}}\ln\frac{V^{C}_{+,HD}(\infty)}{V^{C}_{+,HD}(0)}.  $$

Equation (10) does hold since this is precisely the expression used to estimate *ε*_+,*HD*_ from vaccine RCT information and *ε*_+,*SD*_.

Since all known variables in *f* and *g* have been estimated except for *β* and *p*, we can obtain estimates for *β* and *p* by solving (15) (e.g., numerically).

15 $$ \begin{aligned}  f(\beta,p) = 0,\\ g(\beta,p) =0. \end{aligned}  $$

Solving System (15) yields estimates for *p* and *β*, which we provide with numerical methods in Section “Disease transmission and burden estimates”. Lastly, we note that Equations (9) and (12) remain intact if a latent compartment were to be added to models (1) and (2) to account for the incubation period of influenza.

## Appendix B: Detailed parameter estimation

Here we present the details for the calculations of parameter values *ε*_+,*SD*_,*ε*_+,*HD*_ and *ε*_−,*SD*_ for influenza seasons 2011-2012 and 2012-2013. For this purpose, we utilize an approximation which relates vaccine-modified susceptibility to vaccine effectiveness (VE) [36]. We also leverage influenza vaccine effectiveness (VE) from several studies in the US and Canada.

### 2011-2012 Influenza season

#### Canada

*Informingε*_−,*SD*_: First, we inform *ε*_+,*SD*_ using VE estimates. In Canada, the VE against Real-Time Polymerase Chain Reaction (Real-time PCR) confirmed influenza in a test-negative case-control study was estimated to be 55% among all participants of all ages [25]. We use the approximation *ε*=1−VE to estimate *ε*_−,*SD*_; that is *ε*_−,*SD*_=1−0.55=0.45 [36].

*Informingε*_+,*SD*_: In the same test-negative case-control study, the VE against Real-time PCR confirmed influenza among participants aged 50+ was estimated to be 58% [25]. We use this figure to estimate *ε*_+,*SD*_ using the approximation *ε*=1−VE to find *ε*_+,*SD*_=1−0.58=0.42 [36].

#### US

*Informingε*_−,*SD*_: In the US, the VE against medically-attended influenza among all ages was estimated to be 47% [26]. We now use the approximation *ε*=1−*VE* to estimate *ε*_−,*SD*_ as follows: *ε*_−,*SD*_=1−0.47=0.53.

*Informingε*_+,*SD*_: The VE against medically-attended influenza among those aged 65+ was estimated to be 43% in the US in 2011-2012 [26]. With the relationship *ε*_+,*SD*_=1−*VE* we calculate *ε*_−,*SD*_=0.57 [36].

**Fig. 4:**
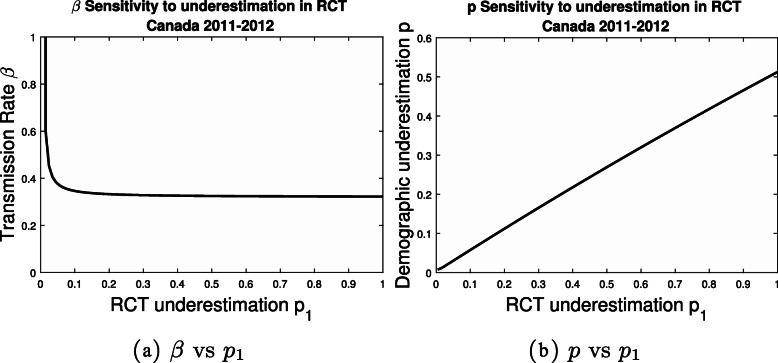
Sensitivity to RCT underestimation, Canada 2011-2012

**Fig. 5:**
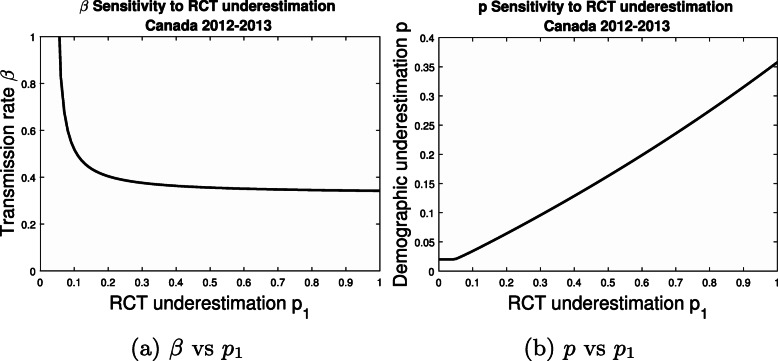
Sensitivity to RCT underestimation, Canada 2012-2013

*Informingε*_+,*HD*_: Lastly, to estimate the high-dose vaccine-modified susceptibility we use Equation (3) in the main text informed with RCT results to calculate *ε*_+,*HD*_/*ε*_+,*SD*_ [17].

$$\frac{\epsilon_{+,HD}}{\epsilon_{+,SD}} = \frac{\log(7230/7253)}{\log(7202/7244)} = 0.55.$$

Note that with the ratio of two vaccine-modified susceptibilities and *ε*_+,*SD*_ known, we can calculate *ε*_+,*HD*_. For the US, we then have *ε*_+,*HD*_=0.55×0.53=0.29.

### 2012-2013

#### Canada

*Informingε*_−,*SD*_: We inform *ε*_−,*SD*_ using the VE against PCR-confirmed influenza from a test-negative case control study in Canada [24]. In this study, the VE against PCR-confirmed influenza was estimated to be 51% [24]. Now, we use the approximation *ε*=1−VE=1−0.51=0.49 [24].

*Informingε*_+,*SD*_: In the same test-negative case control study, VE was estimated to be 47% among participants aged 50+. As a result, we estimate *ε*_+,*SD*_ using the approximation *ε* = 1 − VE to find *ε*_+,*SD*_ = 1 − 0.47 = 0.53 [36].

**Fig. 6:**
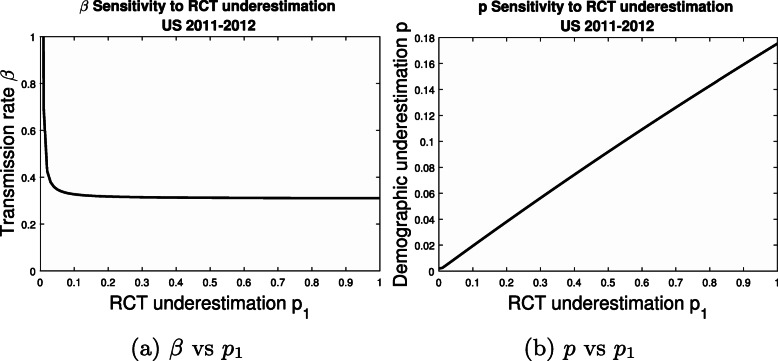
Sensitivity to RCT underestimation, US 2011-2012

**Fig. 7:**
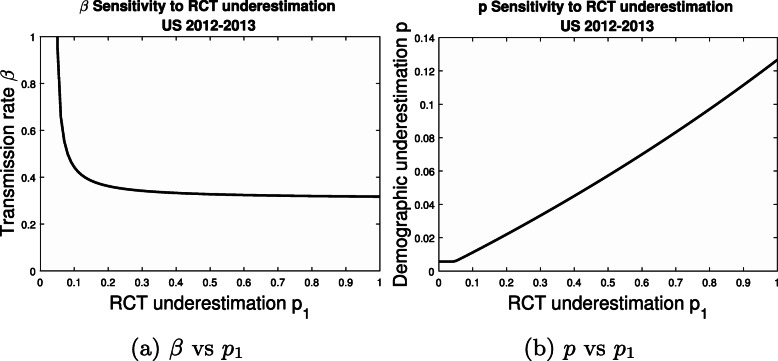
Sensitivity to RCT underestimation, US 2012-2013

#### US

*Informingε*_−,*SD*_: First, we inform *ε*_−,*SD*_ using the estimates for the VE against medically-attended influenza over all ages in the US of 49% [27]. We then calculate *ε*_−,*SD*_=1−0.49=0.51 using the approximation *ε*_+,*SD*_=1−*VE* [36].

*Informingε*_+,*SD*_: In the same US study, VE against medically-attended influenza was estimated to be 29% among those aged 65+ [27]. We then calculate *ε*_+,*SD*_ = 1 − 0.29 = 0.71 using the approximation *ε*_+,*SD*_ = 1−*VE* [36].

*Informingε*_+,*HD*_: Now, to estimate the high-dose vaccine-modified susceptibility we first use Equation (3) in the main text informed by RCT results to calculate *ε*_+,*SD*_/*ε*_+,*HD*_ [17].

$$\frac{\epsilon_{+,HD}}{\epsilon_{+,SD}} = \frac{\log(8532/8737)}{\log(8490/8749)} = 0.79.$$

We use the known ratio: *ε*_+,*SD*_/*ε*_+,*HD*_ to calculate *ε*_+,*HD*_=0.79∗0.71=0.56. In the US we have *ε*_+,*HD*_=0.56.

## Appendix C: Accounting for underestimation in the RCT

In this section, we present the details of the sensitivity analysis exploring the impact of underestimation in the RCT on study results. Specifically, we investigate the sensitivity of *p* and *β* on underestimation in the RCT [17]. To consider underestimation in the RCT, we introduce a underestimation factor *p*_1_ in the RCT. As before, *p* remains the underestimation factor in the general community. We also assume that RCT participants provided with standard-dose and high-dose vaccines are equally underestimated. Lastly, we define *δ* to be the fraction of true remaining vaccinated participants at the end of the RCT.

### Method

The total number of observed infections in the RCT can be written as:

16 $$ {}\bar{I} := V^{C}_{+,SD} (0) + V^{C}_{+,HD} (0) - V^{C}_{+,SD} (\infty) - V^{C}_{+,HD} (\infty).  $$

The above equation can also be formulated in terms of *p*_1_ and *δ*, that is:

17 $$ {}\bar{I} = p_{1} [V^{C}_{+,SD} (0) + V^{C}_{+,HD} (0) - \delta(V^{C}_{+,SD} (\infty) + V^{C}_{+,HD} (\infty))].  $$

The relationship between *p*_1_ and *δ* can be determined by equating (16) and (17) which yields:

18 $$ {}\begin{aligned} \delta &= \frac{1}{V^{C}_{+,SD} (\infty) + V^{C}_{+,HD} (\infty)}\\ &\left[ V^{C}_{+,SD} (0) + V^{C}_{+,HD}(0) - p_{1}^{-1} \left(V^{C}_{+,SD} (0) + V^{C}_{+,HD} (0)\right.\right.\\ &\left.\left.- V^{C}_{+,SD} (\infty) -V^{C}_{+,HD} (\infty) \right) \right]. \end{aligned}  $$

Under these conditions, by conducting the same analysis of model (1) as shown in Appendix A, we derive the condition:

19 $$ \frac{1}{\epsilon_{+,SD}}\ln\frac{\delta V^{C}_{+,SD}(\infty)}{V^{C}_{+,SD}(0)} = \frac{1}{ \epsilon_{+,HD}}\ln\frac{\delta V^{C}_{+,HD}(\infty)}{V^{C}_{+,HD}(0)}.  $$

Continuing to follow the analysis from Appendix A, we arrive at the following system of equations:

20 $$ \begin{aligned} \bar{f}(\beta, p, \delta) &= \delta V^{C}_{+,SD}(\infty) \\ &{}- V^{C}_{+,SD}(0) e^{\frac{\epsilon_{+,SD} \beta}{\gamma} \left(\delta V^{C}_{+,SD}(\infty) - V^{C}_{+,SD}(0) + \delta V^{C}_{+,HD}(\infty) - V^{C}_{+,HD}(0) - p^{-1} \gamma \hat{R}\right)},\\ \bar{g}(\beta,p) &= e^{-(\beta p^{-1}\hat{R}) }\left(S^{O}_{-}(0) + S^{O}_{+}(0)\right) +V^{O}_{-,SD}(0) e^{-(\beta \epsilon_{-,SD} p^{-1}\hat{R})} \\ &\quad+V^{O}_{+,SD}(0)e^{-\left(\beta \epsilon_{+,SD} p^{-1}\hat{R}\right)}+ V^{O}_{+,HD}(0) e^{-\left(\beta \epsilon_{+,HD} p^{-1}\hat{R}\right)}\\ &\quad-(S^{O}_{-}(0)+ S^{O}_{+}(0)+ V^{O}_{-,SD}(0)+V^{O}_{+,SD}(0)+ V^{O}_{+,HD}(0)) +\gamma p^{-1}\hat{R}. \end{aligned}  $$

We analyze the robustness of the estimates of *β* and *p* by varying *δ* in the above system of equations. Exploring sensitivity with respect to *δ* gives insight into how underestimation in the RCT affects our results in Section “Results”. Specifically, for a given *p*_1_, we calculate a corresponding *δ* then solve the above system numerically.

### Results

The relationship between the RCT underestimation factor, *p*_1_, with *β* and *p* estimates is shown in Figs. 4, 5, 6, and 7. We explore the sensitivity of *p*_1_ on *p* and *β* for each context of interest; both regionally in the US and Canada during 2011-12 and 2012-13 influenza seasons. We present the sensitivity analysis using the laboratory-confirmed influenza associated with RI case definition outcomes in the RCT.

Overall, *p* and *β* are robust to underestimation in the RCT. We see that the effect of underestimation in the RCT is negligible on the transmission rate *β* for reasonable values of *p*_1_. On the other hand, *p* is also weakly dependent on *p*_1_. By varying *p*_1_ over its entire range, *p* remains to be on the same order of magnitude.

## Data Availability

Data sharing is not applicable to this article as no datasets were generated or analysed during the current study.

## Abbreviations

SVIR: Susceptible-vaccinated-infected-recovered
RCT: Randomized-controlled trial
PHAC: Public Health Agency of Canada
CDC: Centers for Disease Control and Prevention
SD: Standard-dose vaccine
HD: High-dose vaccine
Real-time PCR: Real-Time Polymerase Chain Reaction
RI: Respiratory illness
